# METNET: a phase II trial of metformin in patients with well-differentiated neuroendocrine tumours

**DOI:** 10.3332/ecancer.2022.1369

**Published:** 2022-03-31

**Authors:** João Glasberg, Aley Talans, Thomás Rivelli Giollo, Débora Zachello Recchimuzzi, João Evangelista Bezerra Neto, Rossana Veronica Mendonza Lopez, Paulo Marcelo Gehm Hoff, Rachel P Riechelmann

**Affiliations:** 1 Department of Clinical Oncology, Instituto do Câncer do Estado de São Paulo, Faculdade de Medicina da Universidade de São Paulo – FMUSP, São Paulo, Brazil; 2 Post Graduate Program, Faculdade de Medicina da Universidade de São Paulo, Brazil; 3 Department of Medical Radiology, Fleury Group, São Paulo, Brazil; 4 Department of Medical Radiology, Hospital Israelita Albert Einstein, São Paulo, Brazil; 5 Center for Translational Research in Oncology, Instituto do Câncer do Estado de São Paulo, Faculdade de Medicina da Universidade de São Paulo – FMUSP, São Paulo, Brazil; 6 Department of Clinical Oncology, AC Camargo Cancer Center, São Paulo, Brazil

**Keywords:** neuroendocrine tumours, metformin, cancer treatment

## Abstract

**Background:**

Preclinical studies have suggested that metformin has anti-tumour effects, likely due to blockage of mammalian target of rapamycin pathway through adenosine monophosphate-activated protein kinase and decreased insulin levels. A retrospective study showed that metformin added to everolimus to treat type 2 diabetes mellitus offered longer progression-free survival (PFS) in patients with pancreatic neuroendocrine tumours (NET).

**Aim(s):**

To evaluate the efficacy and safety of metformin monotherapy in patients with advanced/metastatic well-differentiated NET (WD-NET) of gastroenteropancreatic (GEP) or pulmonary origin.

**Patients and methods:**

Single-arm phase II trial of metformin 850 mg PO twice daily until progression or intolerance for patients with progressive metastatic well-differentiated GEP or pulmonary NET. The primary endpoint was disease control rate (DCR) by RECIST 1.1 at 6 months. Secondary endpoints were response rate, PFS, toxicity and variations in glycaemic profiles (glycaemia, glycated haemoglobin and peptide C and insulin) at baseline, at 30 and 90 days.

**Results:**

From 2014 to 2019, 28 patients were enrolled: median age was 50 years; 84% had non-functional NET, 86% were of GEP origin and 62% had G2 NET. At the time of last follow-up, 26 patients had progression, with 13 (46%) presenting DCR at 6 months and a median PFS of 6.3 months (95% confidence interval: 3.2–9.3). There was no objective response, but one patient with refractory carcinoid syndrome had complete symptom relief, lasting for more than 5 years. Variations in glycaemic profiles were not associated with DCR at 6 months. Diarrhoea was the most common adverse event, being grade 3 or 4 in 10% of the cases.

**Conclusion:**

Metformin monotherapy offers modest anti-tumour activity in well-differentiated GEP or lung NET.

## Introduction

Neuroendocrine tumours (NET) are rare neoplasms originating from neuroendocrine cells present throughout the body. In recent years, there has been an increase in the incidence of NETs, and their current rate of incidence is 6.98 cases per 100,000 individuals in the United States [1]. While this increase can be explained by early-stage disease incidentally detected through imaging and endoscopy tests, the incidence of advanced stage has also increased, with an estimated survival rate of 30% in 5 years [2].

In recent years, several new treatments have emerged to control this heterogeneous group of tumours. The treatments for metastatic NET include peptide receptor radionuclide therapy, somatostatin analogues, chemotherapy, multi-tyrosine kinase inhibitors and everolimus, a potent inhibitor of the mammalian target of rapamycin (mTOR) serine threonine kinase. The choice of electing one of these therapies depends on patients’ clinical features and NET pathological parameters, such as mitotic rate, Ki-67 index, tumour volume and primary tumour site, making the treatment of NET a challenge [3].

The mTOR blockade leads to inhibition of the Phosphoinositide 3-kinase/AKT (PI3K/AKT)/mTOR pathway, which plays an important role in cell growth, protein translation, and cell metabolism in several types of neoplasms [4]. For example, a preclinical study in pancreatic NET (PNET) cell cultures demonstrated that mTOR inhibition by rapamycin reduced cell proliferation and induced apoptosis [5]. Thereafter, a series of placebo-controlled randomised clinical trials with everolimus in different populations of NET patients confirmed the anti-tumour effects of the mTOR inhibition in NET, as demonstrated by significant gains in progression-free survival (PFS) times [6–8]. Another characteristic of everolimus is its role in reducing hormonal secretion of functioning tumours such as metastatic insulinoma and NET patients with carcinoid syndrome [6, 9].

Metformin is widely used to treat individuals with type 2 diabetes mellitus (T2DM). Epidemiological studies in patients with T2DM suggest a possible protective role of metformin against the development of neoplasms [10, 11]. In addition, preclinical studies have shown that metformin reduced cell viability in neuroendocrine cell cultures [12,13], mainly through mTOR inhibition [14-16]. Clinical evidence has also pointed to anti-tumour properties of metformin. The larger retrospective series evaluated 445 Italian patients with metastatic pancreatic NET treated with everolimus with or without somatostatin analogues or somatostatin analogues alone. In this study, investigators reported a twice longer median PFS for patients who were administered metformin to treat T2DM, when compared with patients who received insulin, and a significant reduced risk of progression in comparison to normoglycaemic patients: median of 44.2 months for those treated with metformin [hazard ratio (HR): 0.81; 95% confidence interval (CI): 0.60–1.1; *p* = 0. 18 versus normoglycaemic patients] and 20.8 months for those treated with others treatments (hazard ratio, 0.49; 95% confidence interval, 0.34–0.69; *p* < 0.0001) [17]. Yet, it remains to be determined whether metformin has anti-tumour activity in non-diabetic NET patients. While the mechanisms by which metformin exerts its anti-tumour effect are still not completely elucidated, studies have indicated two main mechanisms of action: (1) inhibition of mTOR, via the activation of adenosine monophosphate-activated protein kinase, which is a negative modulator of mTOR [18–22]; and (2) reduction of promitogenic factors such as insulin and insulin-like growth factor 1 (IGF-1), a known metformin effect on glucose metabolism [19].

Based on such preliminary evidence of NET-directed metformin, we conducted a phase II single-arm clinical trial to investigate the anti-tumour activity of metformin monotherapy in patients with advanced/metastatic well-differentiated NET (WD-NET) of gastroenteropancreatic (GEP) or pulmonary origin.

## Methods – patients

The study participants were recruited from the gastrointestinal outpatient cancer clinic of the Instituto do Cancer do Estado de São Paulo, São Paulo, which is publicly funded, affiliated with the University of Sao Paulo and among the largest cancer centres in Brazil. The local ethics committee approved the study and all patients provided written informed consent. The trial was registered in ClinicalTrials.gov as NCT02279758.

Eligible patients were older than 16 years, had a histological diagnosis of a WD grade 1 or 2 NET of GEP or pulmonary origin and metastatic disease, were Eastern Cooperative Oncology Group (ECOG) score of 0–2 and presented radiological progression of disease within the previous 3 months as per the attending physician’s discretion. WD-NET was defined based on the WHO classification 2019 grade 1 and 2 for digestive NEN [23]; patients with typical or atypical lung NEN were also eligible. Any prior line of therapy was allowed, and somatostatin analogues could be used concurrently with metformin to control hormone-related symptoms of functioning NET. Exclusion criteria were inability to take oral tablets, clinically relevant laboratory abnormalities (haemoglobin < 8 g/dL, platelets < 100,000/mm^3^, neutrophil count < 1,500/mm^3^ and creatinine > 1.5 times the upper limit of normal range), decompensated hormonal syndrome or rapidly progressive disease requiring cytotoxic chemotherapy, current use of metformin or used this drug in the last 3 months prior to study enrolment, history of hypersensitivity to metformin, active infection, use of experimental therapies or simultaneous participation in another clinical trial, chemotherapy within 3 weeks of study enrolment and pregnant or lactating patients.

## Methods – materials

In this single-institution, single-arm phase II clinical trial, we enrolled patients with WD-NET to receive metformin as a single agent until disease progression, unacceptable toxicity or consent withdrawal. Metformin was administered in 850 mg PO tablets twice a day with water and meals as monotherapy for patients with non-functioning tumours and in combination with somatostatin analogues for functioning NETs. In case of grade 3 or 4 toxicity, according to the National Cancer Institute Common Terminology Criteria for Adverse Events criteria (NCI-CTC AE) version 4.03, a dose reduction of metformin to 850 mg once daily was allowed after improvement of symptoms to grade 1. Patients who required more than 3 weeks to recover from drug-related adverse events or who could not tolerate metformin 850 mg per os (PO) once daily were discontinued from the trial – but continued to be followed with image tests to evaluate disease progression. Metformin was provided to patients by the study team at every medical visit. Drug adherence was monitored on every medical visit by the trial investigators, directly enquiring patients and performing drug accountability by checking empty blisters. We expected a minimal of 80% of the adherence of metformin throughout the study.

After signing the informed consent, patients underwent the following baseline assessments: radiographic evaluation with computerised tomography (CT) and/or magnetic resonance imaging of thorax, abdomen and pelvis within 4 weeks of metformin first dose; clinical examination and consultation, serum routine laboratory tests (complete blood cell counts, sodium, potassium, urea, creatinine, aspartate aminotransferase and alanine aminotransferase), specific hormones/peptides for functioning NET and fasting glycaemic profile (fasting blood glucose, serum insulin, glycosylated haemoglobin and peptide C within 7 days of metformin first dose). Patients underwent medical examination and serum tests monthly (±7 days) and radiographic evaluation and specific hormones/peptides for functioning NET every 3 months (±7 days) until disease progression. Fasting glycaemic profiles were collected at baseline, in 3 and 6 months from metformin first dose.

The following demographics, clinical and NET-related data, were prospectively collected: age at study entry, sex, primary NET origin, if functioning NET (and related hormone/peptide), comorbid illnesses, concurrent medications, ECOG, weight and height, NET type (G1 or G2) and Ki-67 index.

## Methods – endpoints and assessments

The primary endpoint of the trial was disease control rate (DCR) at 6 months from metformin initiation. DCR was defined as the proportion of patients without tumour progression (e.g., stable, partial or complete response), according to the response evaluation criteria in solid tumours (RECIST) 1.1 [24]. Secondary endpoints were PFS, toxicity measured by the NCI-CTC AE version 4.03 and variations in glycaemic profiles throughout the study and their association with DCR at 6 months. PFS was defined as the time from first metformin dose to documented disease progression or death, according to the RECIST 1.1 [24]. We planned exploratory analyses of PFS and DCR by subgroups of body mass index (BMI <25 and ≥25 kg/m^2^). The use of this cut-off was based on BMI scale, which is considered normal BMI between 18.5 and 24.9 kg/m^2^.

## Methods – statistical analysis

Descriptive statistics were used to summarises categorical and quantitative variables, with their respective measures of dispersion. Comparisons between quantitative variables were made using the Mann–Whitney’s non-parametric test. Efficacy and toxicity analyses were performed by intent to treat, considering all patients who received at least one dose of metformin. 95% confidence intervals (CI) were calculated for the proportions of patients who achieved DCR. The Kaplan–Meier method was used to estimate PFS of all patients and by subgroups of body mass index (BMI <25 and ≥25 kg/m^2^). The reverse Kaplan–Meier method was used to estimate the follow-up time, considering date of progression as the outcome event. The comparison between such subgroups was made by the log-rank test. Associations between BMI subgroups and DCR at 6 months were evaluated by the Chi-square test. Associations between variations in fasting glycaemic profiles and DCR at 6 months were measured by Mann–Whitney’s test. Two-sided *p*-values < 0.05 were considered significant. The best response achieved by each patient, reported as percentages of maximum changes in target NET lesions sizes from baseline (excluding new lesions), was presented in a waterfall plot.

The sample size was arbitrarily determined to be 30 patients, since this was the first prospective study addressing the efficacy of metformin for NET. For interpretation purposes, the study would be considered positive if at least half of the patients achieved the primary outcome of DCR at 6 months. This was based on the results of the phase III trials in NET, where approximately half of the patients are progression-free at 6 months [7, 8].

## Results

From July 2014 to August 2019, 29 patients were offered the trial and 28 consented, composing the study population; one patient refused to participate because she was concerned with the possible metformin-associated side effects of weight loss. The majority (53%) were male, with a median age of 50 years and median BMI of 27.05 kg/m^2^. Two (7%) patients had T2DM and both were taking insulin. The most common primary sites were midgut (*N* = 12; 43%) and pancreas (*N* = 9; 32%). Twenty-four (85%) patients had non-functioning NET and most had a grade 2 NET (*n* = 20; 71%). Of these grade 2 NET patients, 12 had Ki-67 between 3% and 10%, and 8 patients had Ki-67 between 11% and 20%. The great majority of patients (*N* = 21; 75%) received at least one prior systemic NET-directed therapy prior to metformin, with nearly 40% being pretreated with a somatostatin analogue. Four patients (14.2%) had functioning NETs and received metformin combined with a somatostatin analogue. Table 1 summarises the patients’ characteristics.

**Figure 1.**
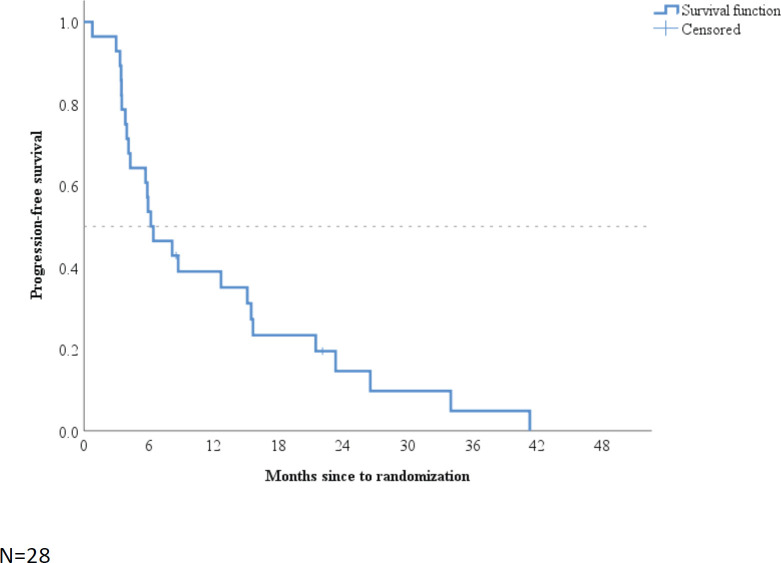
Kaplan–Meier plot of progression-free survival.

Thirteen patients achieved disease control at 6 months, with a DCR of 46% (95% CI: 27–66%). There was no association between DCR at 6 months and BMI subgroups (p=0.48). In a median follow-up time of 6.3 months (95% CI: 2.5–10.1), median PFS was 6.2 months (95% CI: 3.2–9.3; Figure 1).

**Figure 2.**
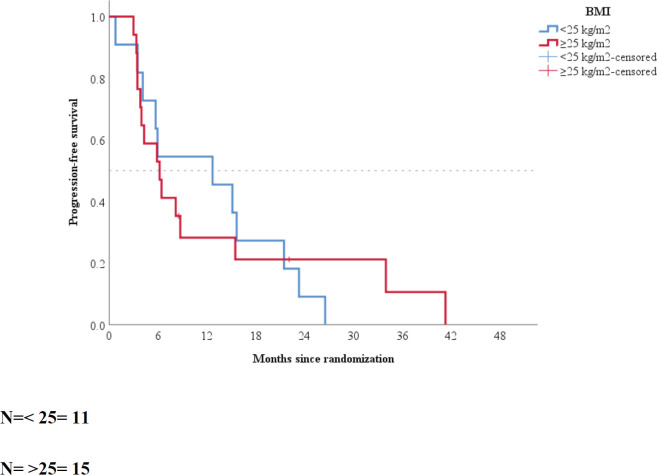
Kaplan–Meier plot according to baseline body mass index.

When patients were grouped according to BMI, the median PFS of patients with BMI of <25 and ≥25 kg/m^2^ was 12.9 (95% CI: 2.5–23.22) and 6.3 (95% CI: 3.38–9.21) months, respectively (*p* = 0.93; Figure 2).

**Figure 3.**
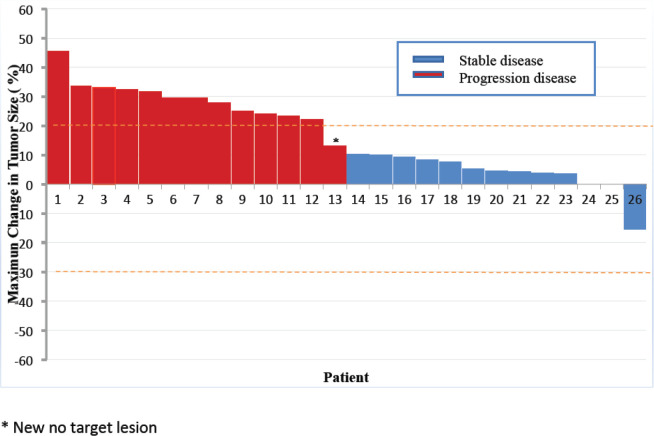
Waterfall plot.

No patient presented objective radiological response. Stable disease was the best response achieved by 86% of the patients. Only four patients had functioning tumours, with three presenting carcinoid syndrome. No reduction in 5-hydroxyindoleacetic acid levels was observed in the 24-hour urine test with the use of metformin. However, one patient with refractory carcinoid syndrome to octreotide LAR 30 mg reported remission of symptoms with the use of metformin combined with octreotide, lasting for 3.3 years. Figure 3 shows a summary of the maximum changes in tumour sizes from baseline.

### Glycaemic profiles

Median and mean serum levels of fasting insulin, glucose, glycated haemoglobin and C-peptide at baseline, at 3 months and 6 months, are shown in Table 2.

No significant association was observed between variations in serum levels of fasting glycaemic profiles and DCR at 6 months (insulin, *p* = 0.54; blood glucose, *p* = 0.33; glycated haemoglobin, *p* = 0.069; C-peptide, *p* = 0.68). Table 3 shows the median and mean baseline glycaemic profile of patients who achieved DCR in 6 months versus those who did not.

**Figure 4.**
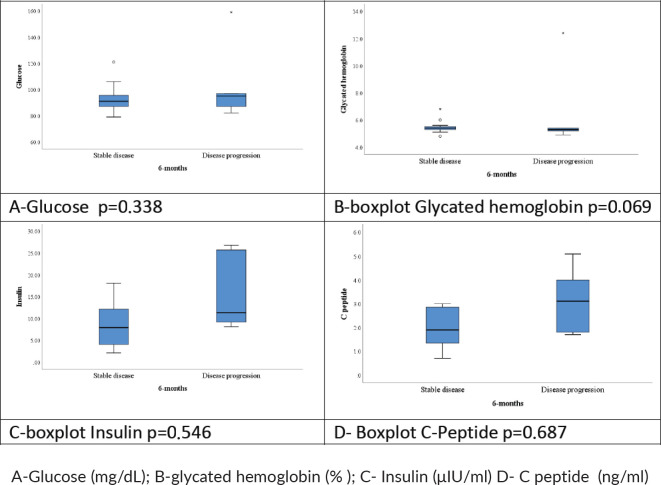
Boxplot of fasting glycemic profiles.

Figure 4 shows box plots of fasting glycaemic profiles according to DCR at 6 months: disease stabilisation/response or tumour progression.

### Toxicity

Metformin at a dose of 850 mg twice a day was generally well tolerated. The most prevalent toxicity was diarrhoea. Grade 1 or 2 was found in 21% of the patients and grade 3 or 4 in 14%. Four (14%) patients required dose reduction. All of them are related to diarrhoea grade 3 or 4. The use of metformin was suspended until the control of the symptoms to grade 1; then, it was reintroduced in one tablet per day. There is no patient who discontinued the treatment due to toxicity (Table 4). All patients achieved the minimum of 80% of drug adherence.

## Discussion

This phase II trial, unlike preclinical and retrospective clinical studies in patients with metastatic WD NET [14, 15, 17, 25, 26], did not confirm the anti-tumour effects of metformin in patients with grade 1 or 2 NETs. Nearly two-thirds (64%) of the patients presented disease progression before completing 6 months of treatment, no patient experience radiological response and the median PFS was only 6.3 months, similar to what has been observed with placebo in randomised phase III trials. To explore predictive factors of DCR, variations in the glycaemic profile of patients from baseline to 6 months and baseline BMI were analysed but no associations were observed.

Despite the small sample size of our trial, the results presented here indicate a clear lack of tumour activity of metformin monotherapy in WD-NETs. Opposite results were reported in preclinical studies. Vlotides *et al* [15] studied metformin activity in three cell lines of NET: in three human tumour cell lines (BON1 human pancreatic NET cells, bronchopulmonary NCI‑H727 and midgut GOT1); increasing concentrations of metformin (0.1–10 mM) led to inhibition of cell viability in all three cell populations [15]. It is possible that the dose used in our trial, the label dose of metformin for T2DM, is not enough to induce cancer-directed effects as metformin concentrations achieved in an *in vitro* study is much higher than what is achieved *in vivo* (1–20 mM versus 18 µM) [27].

Based on the data obtained from an *in vitro* study that indicated a possible mTOR inhibition activity of metformin, there was increased interest in the study of metformin and everolimus. It is believed that this association could play a synergistic role in enhancing mTOR inhibition by everolimus, inhibiting a possible resistance mechanism to this drug. An *in vitro* study evaluated the association of metformin and everolimus in cell cultures taken from patients with PNETs and pulmonary NETs. In the PNET culture, the combination of metformin and everolimus was more effective in inhibiting cell proliferation than each of them administered individually [14]. The combination did not inhibit proliferation in pulmonary NET cells [14]. Clinical evidence of metformin in NET is mostly from retrospective studies and also in patients treated with everolimus [17, 25, 26]. The largest retrospective study, the PRIME-NET, analysed 445 PNETs patients treated in 24 Italian medical centres. In that study, all patients received everolimus with or without a somatostatin analogue. Of all patients, 236 developed T2DM: 112 were treated with metformin and 124 with other hypoglycaemic drugs, including insulin. The use of metformin among patients with T2DM, in comparison with those treated with other treatments, was associated with an improvement in median PFS from 20.8 to 44.2 months, with a 51% reduction in the risk of progression or death when compared to normoglycaemic patients (HR: 0.49; 95% CI: 0.34–0.69; *p* < 0.00001) [26]. Another study, conducted in France, retrospectively evaluated NET patients treated with metformin and everolimus. A total of 213 patients received everolimus, and of these, only 48 had baseline T2DM (19 received metformin). No significant difference in median PFS was noted in the group treated with metformin and everolimus: 15.24 months versus 9.0 months for everolimus alone (HR 0.87, 95% CI: 0.54–1.39; *p* = 0.55) [26].

Unfortunately, everolimus is not reimbursed for NET in the Brazilian public health system, where all patients were treated, which prevented us from exploring metformin co-administered with everolimus in a prospective study.

To date, all studies that have clinically evaluated metformin activity in NET are retrospective. Therefore, the studied population comprised patients with T2DM [17, 25, 26]. The association between T2DM and an increased incidence of cancer has already been demonstrated by several studies [11, 28]. It is believed that the insulin resistance in patients with T2DM would lead to hyperinsulinemia, which would stimulate multiple cell signalling cascades and growth factors. A recent meta-analysis that collected data from 20 publications with a total of 13,008 patients with T2DM and various cancer types showed that the use of metformin was associated with improvement in overall survival and cancer-specific survival (HR: 0.66; 95% CI: 0.55–0.79 and HR: 0.62; 95% CI: 0.46–0.84, respectively) [29], suggesting that patients with T2DM and cancer should be treated with metformin. Herrera-Martínez et al. retrospectively analysed two cohorts of patients with NETs (gastrointestinal NETs and pulmonary carcinoid). Patients with both T2DM and pulmonary carcinoid had more advanced diseases, larger tumours, higher pleural invasion rates and higher death rates [16]. In our study, we had two patients with T2DM and they experienced disease progression before 6 months of treatment.

The supposed anti-tumour effect of metformin via reduction of promitogenic factors such as hyperinsulinemia and increased IGF-1 led to the hypothesis that variations in fasting glycaemic profiles could be a predictor of metformin activity. Yet, we did not observe associations between variations in glycaemic profiles at baseline and at DCR at 6 months. In the PRIME-NET study, fasting baseline blood glucose levels were also not associated with PFS [17].

BMI was another factor explored in the present study. It is believed that fat would play a proinflammatory role and increase insulin resistance, ultimately leading to an increase in signalling pathways and cell proliferation [30]. In the present study, BMI was not found to be a predictor of DCR. The possible association between obesity and NET was analysed in a case/control study with 96 cases and 96 control subjects, and no association was noted between BMI and NET [31]. Similarly, in another retrospective analysis of 324 patients treated at a Swedish institution, BMI of ≥25 kg/m^2^ was not found to predict a worse prognosis in PNET patients [32]. A similar result was found in the analysis of patients with resected PNET, in which BMI did not correlate with a higher rate of recurrence or mortality [21].

The limitations of this trial should be mentioned. This was a single-centre, uncontrolled phase II trial, which compromises its external validity. To improve internal validity, we enrolled patients with documented progressive disease. Another limitation of this study was not achieving the target sample of 30 patients planned. However, despite its limitations, our study did not identify anti-proliferative signals of metformin in NET, even when noticing that our patients were not heavily pretreated. Therefore, we do not think that further trials of metformin monotherapy are required to confirm our findings. Conversely, the evaluation of cancer-directed effects of metformin combined with everolimus should be sought. As an example, a single-arm phase II trial (NCT02294006) is recruiting patients to investigate the anti-proliferative potential of metformin with everolimus and octreotide LAR in NETs patients.

## Conclusion

In conclusion, the use of metformin monotherapy did not demonstrate clinically significant anti-tumour effects in patients with well-differentiated grade 1 and 2 NET. We think that further trials of metformin combined with everolimus, in patients with or without related hyperglycaemia, are worth being conducted.

## Funding

This research did not receive any specific grant from any funding agency in the public, commercial or not-for-profit sector.

## Authors’ contributions

Conception and design: J Glasberg and R P Riechelmann. Collection and assembly of data: J Glasberg, A Talans, T R Giollo, R Canella and J E Bezerra Neto. Data analysis and interpretation: J Glasberg, R P Riechelmann and R V Mendonza Lopez. Manuscript writing: J Glasberg and R P Riechelmann. Final approval of manuscript: J Glasberg, A Talans, T Rivelli Giollo, R Canella, J E Bezerra Neto, P M Glenh Hoff and R P Riechelmann.

## Conflicts of interest

R P Riechelmann: honoraria for lectures from Novartis.

J Glasberg, A Talans, T Rivelli Giollo, D Zachello Recchimuzzi, J E Bezerra Neto, R V Mendonza Lopez and P M Gehm Hoff: none.

## Figures and Tables

**Table 1.** Characteristics of the study population.

Characteristic	Value
Age, years	50 (32–72)
Gender	
Male	15 (53.6)
Female	13 (46.4)
BMI (kg/m	
<25	11 (39.3)
≥25	17 (60.7)
ECOG	
0	21 (75)
1	6 (21.4)
2	1 (3.6)
Comorbidity	
T2DM	2 (7.1)
Depression	5 (17.8)
Hypothyroidism	3 (10.7)
HPB	3 (10.7)
Primary tumour site	
Rectum	1 (3.6)
Unknown	2 (7.1)
Lungs	4 (14.3)
Pancreas	9 (32.1)
Midgut	12 (43.9)
Functional NET	4 (14.2)
Carcinoid syndrome	3 (10.7)
Cushing syndrome	1 (3.5)
Tumour grade	
NET G1	8 (28.6)
NET G2	20 (71.4)
Treatments performed before study entry	21 (75)
Number of treatments	
1	11 (39.3)
2	5 (17.8)
3	4 (14.3)
4	0
5	1(3.6)
Somatostatin analogues	13(46.4)
Interferon-alpha	2 (7.1)
^177^Lu-DOTATATE	1 (3.6)
TACE/TAE	2 (7.1)
Chemotherapy	9 (32.1)
Debulking surgery	1 (3.6)

Data are presented median and range or *n* (%).

ECOG PS= Eastern Cooperative Oncology Group performance status, T2DM = Diabetes mellitus type 2, HPB = High blood pressure, TACE = Transarterial chemoembolisation, TAE = transarterial embolisation, ^177^Lu-DOTATATE = ^177^Lu-tetraazacyclododecanetetraacetic acid-octreotide

**Table 2.** Fasting glycaemic profile.

Fasting glycaemic test	Median	Mean
Glucose (mg/dL)		
Baseline	89 (75–302)	99.64 ± 41.4
90 days	86 (64–162)	89.2 ± 20.1
180 days	93 (79–159)	96.7 ± 18.7
HbA1 (%)		
Baseline	5.45 (4.8–11.5)	6.0 ± 1.5
90 days	5.40 (4.3–9.8)	5.7 ± 1,1
180 days	5.40 (4.8-12.4)	5.8 ± 1.7
Insulin (μIU/mL)		
Baseline	9.5 (2.0–23.2)	10.7 ± 5.6
90 days	7.30 (1.6–23.3)	8.9 ± 4.9
180 days	10.10 (2.1–26.8)	10.81 ± 7.1
C-Peptide (ng/mL)		
Baseline	2.4 (0.32–4.8)	2.3 ± 1.1
90 days	1.9 (0.1–5.5)	2.2 ± 1.1
180 days	2.07 (0.7–5.1)	2.3 ± 1.1

Data are presented as median (range) and mean ± SD

HbA1 = glycosylated haemoglobin

**Table 3.** Glycaemic profiles during the trial.

Fasting glycaemic test	Baseline fasting glycaemic profile	
Glucose (mg/dL)	Median	Mean
Stable disease	91 (75–129)	95.9 ± 14.3
Disease progression	88 (78–302)	102 ± 55.8
HbA1 (%)		
Stable disease	5.4 ( 4.9–9.0)	5.88 ± 1.2
Disease progression	5.5 ( 4.8–11.5)	6.12 ± 1.8
Insulin (μIU/mL)		
Stable disease	7.7 (2–19.1)	8.8 ± 5.4
Disease progression	12.5 (5.5–23.2)	12.3 ± 5.5
C-Peptide (ng/ml)		
Stable disease	2.2 (0.5–2.8)	1.87 ± 0.7
Disease progression	2.6 ( 0.32–4.8)	2.7 ± 1.3

Data are presented as median (range) and mean ± SD

HbA1 = glycosylated haemoglobin

**Table 4.** Treatment-related adverse events.

Toxicity	*N*(%)
Nausea	
G1-2	6 (21.4)
G3-4	0
Diarrhoea	
G1-2	6 (21.4)
G3-4	4 (14.3)
Vomiting	
G1-2	1 (3.6)
G3-4	0
Dyspepsia	
G1-2	3 (10.8)
G3-4	0
Abdominal pain	
G1-2	4 (14.3)
G3-4	0
Xerostomia	
G1-2	1 (3.6)
G3-4	0
Hyporexia	
G1-2	2 (7.2)
G3-4	0

Data are presented as *n* (%)

## References

[1] Dasari A, Shen C, Halperin D (2017). Trends in the incidence, prevalence, and survival outcomes in patients with neuroendocrine tumors in the United States. JAMA Oncol.

[2] Halfdanarson TR, Rabe KG, Rubin J (2008). Pancreatic neuroendocrine tumors (PNETs): incidence, prognosis and recent trend toward improved survival. Ann Oncol.

[3] Riechelmann RP, Weschenfelder RF, Costa FP (2017). Guidelines for the management of neuroendocrine tumours by the Brazilian gastrointestinal tumour group. Ecancermedicalscience.

[4] Meric-Bernstam F, Gonzalez-Angulo AM (2009). Targeting the mTOR signaling network for cancer therapy. J Clin Oncol.

[5] Chiu CW, Nozawa H, Hanahan D (2010). Survival benefit with proapoptotic molecular and pathologic responses from dual targeting of mammalian target of rapamycin and epidermal growth factor receptor in a preclinical model of pancreatic neuroendocrine carcinogenesis. J Clin Oncol.

[6] Pavel ME, Hainsworth JD, Baudin E (2011). Everolimus plus octreotide long-acting repeatable for the treatment of advanced neuroendocrine tumours associated with carcinoid syndrome (RADIANT-2): a randomised, placebo-controlled, phase 3 study. Lancet.

[7] Yao JC, Shah MH, Ito T (2011). Everolimus for advanced pancreatic neuroendocrine tumors. N Engl J Med.

[8] Yao JC, Fazio N, Singh S (2016). Everolimus for the treatment of advanced, non-functional neuroendocrine tumours of the lung or gastrointestinal tract (RADIANT-4): a randomised, placebo-controlled, phase 3 study. Lancet.

[9] Matej A, Bujwid H, Wronski J (2016). Glycemic control in patients with insulinoma. Hormones (Athens).

[10] Dowling RJ, Goodwin PJ, Stambolic V (2011). Understanding the benefit of metformin use in cancer treatment. BMC Med.

[11] Vigneri P, Frasca F, Sciacca L (2009). Diabetes and cancer. Endocr Relat Cancer.

[12] Quinn BJ, Kitagawa H, Memmott RM (2013). Repositioning metformin for cancer prevention and treatment. Trends Endocrinol Metab.

[13] Miranda VC, Barroso-Sousa R, Glasberg J (2014). Exploring the role of metformin in anticancer treatments: a systematic review. Drugs Today (Barc).

[14] Vitali E, Boemi I, Tarantola G (2020). Metformin and everolimus: a promising combination for neuroendocrine tumors treatment. Cancers (Basel).

[15] Vlotides G, Tanyeri A, Spampatti M (2014). Anticancer effects of metformin on neuroendocrine tumor cells in vitro. Hormones (Athens).

[16] Herrera-Martinez AD, Pedraza-Arevalo S, L-López F (2019). Type 2 diabetes in neuroendocrine tumors: are biguanides and statins part of the solution?. J Clin Endocrinol Metab.

[17] Pusceddu S, Vernieri C, Di Maio M (2018). Metformin use is associated with longer progression-free survival of patients with diabetes and pancreatic neuroendocrine tumors receiving everolimus and/or somatostatin analogues. Gastroenterology.

[18] Pierotti MA, Berrino F, Gariboldi M (2013). Targeting metabolism for cancer treatment and prevention: metformin, an old drug with multi-faceted effects. Oncogene.

[19] Pernicova I, Korbonits M (2014). Metformin--mode of action and clinical implications for diabetes and cancer. Nat Rev Endocrinol.

[20] Daugan M, Dufay Wojcicki A, Hayer B (2016). Metformin: an anti-diabetic drug to fight cancer. Pharmacol Res.

[21] Cherenfant J, Stocker SJ, Gage MK (2013). Predicting aggressive behavior in nonfunctioning pancreatic neuroendocrine tumors. Surgery.

[22] Zi F, Zi H, Li Y (2018). Metformin and cancer: an existing drug for cancer prevention and therapy. Oncol Lett.

[23] Pelosi G, Sonzogni A, Harari S (2017). Classification of pulmonary neuroendocrine tumors: new insights. Transl Lung Cancer Res.

[24] Eisenhauer EA, Therasse P, Bogaerts J (2009). New response evaluation criteria in solid tumours: revised RECIST guideline (version 1.1). Eur J Cancer.

[25] Pusceddu S, Buzzoni R, Vernieri C (2016). Metformin with everolimus and octreotide in pancreatic neuroendocrine tumor patients with diabetes. Future Oncol.

[26] Hue-Fontaine L, Lemelin A, Forestier J (2020). Metformin and everolimus in neuroendocrine tumours: a synergic effect?. Clin Res Hepatol Gastroenterol.

[27] Tucker GT, Casey C, Phillips PJ (1981). Metformin kinetics in healthy subjects and in patients with diabetes mellitus. Br J Clin Pharmacol.

[28] Giovannucci E, Harlan DM, Archer MC (2010). Diabetes and cancer: a consensus report. Diabetes Care.

[29] Yin M, Zhou J, Gorak EJ (2013). Metformin is associated with survival benefit in cancer patients with concurrent type 2 diabetes: a systematic review and meta-analysis. Oncologist.

[30] Lee DH, Giovannucci EL (2019). The obesity paradox in cancer: epidemiologic insights and perspectives. Curr Nutr Rep.

[31] Santos AP, Santos AC, Castro C (2018). Visceral obesity and metabolic syndrome are associated with well-differentiated gastroenteropancreatic neuroendocrine tumors. Cancers (Basel).

[32] Ekeblad S, Skogseid B, Dunder K (2008). Prognostic factors and survival in 324 patients with pancreatic endocrine tumor treated at a single institution. Clin Cancer Res.

